# Physical exercise at the workplace prevents deterioration of work ability among healthcare workers: cluster randomized controlled trial

**DOI:** 10.1186/s12889-015-2448-0

**Published:** 2015-11-25

**Authors:** Markus D. Jakobsen, Emil Sundstrup, Mikkel Brandt, Kenneth Jay, Per Aagaard, Lars L. Andersen

**Affiliations:** National Research Centre for the Working Environment, Lersø Parkalle 105, Copenhagen, Denmark; Muscle Physiology and Biomechanics Research Unit, Institute of Sports Science and Clinical Biomechanics, University of Southern Denmark, Odense, Denmark; Physical Activity and Human Performance group, SMI, Department of Health Science and Technology, Aalborg University, Aalborg, Denmark; Electronics and Computer Science, Faculty of Physical and Applied Sciences, University of Southampton, Southampton, UK

**Keywords:** Musculoskeletal disorders, Occupational health, Health care, Strength training, Back pain

## Abstract

**Background:**

Imbalance between individual resources and work demands can lead to musculoskeletal disorders and reduced work ability. The purpose of this study was to investigate the effect of workplace- versus home-based physical exercise on work ability among healthcare workers.

**Methods:**

Two hundred female healthcare workers (Age: 42.0, BMI: 24.1, work ability index [WAI]: 43.1) from 18 departments at three Danish hospitals participated (Copenhagen, Denmark, Aug 2013—Jan 2014). Participants were randomly allocated at the cluster level to 10 weeks of: 1) workplace physical exercise (WORK) performed during working hours for 5x10 min per week and up to 5 group-based coaching sessions on motivation for regular physical exercise, or 2) home-based physical exercise (HOME) performed during leisure time for 5x10 min per week. Both groups received ergonomic counseling on patient handling and use of lifting aides. The main outcome measure was the change from baseline to 10-week follow-up in WAI.

**Results:**

Significant *group by time* interaction was observed for WAI (*p* < 0.05). WAI at follow-up was 1.1 (0.3 to 1.8) higher in WORK compared with HOME corresponding to a small effect size (Cohens’d = 0.24). Within-group changes indicated that between-group differences were mainly caused by a reduction in WAI in HOME. Of the seven items of WAI, item 2 (work ability in relation to the demands of the job) and item 5 (sickness absence during the past year) were improved in WORK compared with HOME (*P* < 0.05).

**Conclusions:**

Performing physical exercise together with colleagues at the workplace prevents deterioration of work ability among female healthcare workers.

**Trial registration number:**

ClinicalTrials.gov NCT01921764. Registered 10 August 2013.

## Background

Work ability reflects the interaction between the individual’s resources (e.g., health, competence and know-how and physical and mental capacity) and the specific demands of the work task(s) [1]. Impairments in work ability have been associated with musculoskeletal pain, chronic disease, sickness absence, early retirement and all-cause mortality [2–7]. Moreover, individuals exposed to forceful and awkward working postures and reduced lack of recovery have an elevated risk of musculoskeletal disorders and impaired work ability [8–12].

Healthcare work involves frequent exposure to risk factors for back pain such as sudden and high loadings, including spinal twisting and bending during patient handling [13, 14]. Although the implementation of manual handling equipment has increased the preventive efforts in the healthcare sector the incidences of musculoskeletal pain remains high [15]. Accordingly, effective initiatives for preventing this imbalance between exposure and individual capacity from critically impairing work ability among healthcare workers are needed. Moreover, in addition to the emerging global shortage in the healthcare workforce [16], associations between low work ability and intention to quit the healthcare workforce highlights the importance of increasing (or sustaining) the individual resources of the healthcare worker [17].

Several intervention studies conducted in different occupational settings have investigated the effect of physical exercise on work ability, however predominantly with little or no effect [18–22]. Conversely, Pohjonen & Ranta was able to demonstrate that 9 months of supervised exercise intervention, twice a week at the workplace, was sufficient in preventing a decline in work ability after 1 and 5 years in female care aides [21]. Nevertheless, low adherence and high drop-out rates in most of the aforementioned studies highlights the difficulties of implementing effective interventions at the workplace. Consequently, high qualitative randomized controlled trials investigating the effects of physical exercise on work ability are warranted.

Although supervised and group-based intervention protocols seem to enhance exercise adherence compared to home-based exercise interventions [23–25] effective interventions at the workplace can be very costly. Nevertheless, some employees might disfavor exercising with their colleagues at work whereas others find it more motivating to exercise with colleagues and exercise instructors at the workplace. Altogether, a need exists to examine if increasing the healthcare workers individual capacity by means of physical exercise can increase work ability. Secondly, we need to know if physical exercise performed as a low cost home-based intervention is equally effective as a workplace based intervention that invests working hours, equipment and on-site training instructors.

The aim of the present study, therefore, was to investigate the effect of workplace-based versus home-based physical exercise on work ability among healthcare workers. We tested the null-hypothesis that supervised physical exercise at the workplace and home-based exercise will not have different effects on work ability.

## Methods

### Study design

We conducted a two-armed parallel-group, single-blind, cluster randomized controlled trial with allocation concealment among female healthcare workers recruited from three hospitals (18 departments) situated in Copenhagen, Denmark, from August 2013 to January 2014. As each hospital department functions as a separate entity, cluster randomization at the department level was chosen to increase adherence and avoid contamination between interventions. The participants were allocated to a 10-week intervention period and randomly assigned to receive either workplace or home-based physical exercise. To ensure that the study aim, hypothesis, and primary outcome parameters were pre-defined the study was approved by The Danish National Ethics Committee on Biomedical Research (Ethical committee of Frederiksberg and Copenhagen; H-3-2010-062) and registered in ClinicalTrials.gov (NCT01921764) prior to enrolment of participants. The study adhered to the CONSORT checklist to ensure transparent and standardized reporting of the trial. All experimental conditions conformed to The Declaration of Helsinki. The study protocol and primary outcome (change in average muscle pain intensity of the low back, neck and shoulder) has been published elsewhere [26, 27].

### Recruitment and randomization

The recruitment of participants was two-phased and consisted of a short screening questionnaire conducted in June 2013, followed by a baseline clinical examination and questionnaire performed in Aug-Sept 2013.

**Fig. 1:**
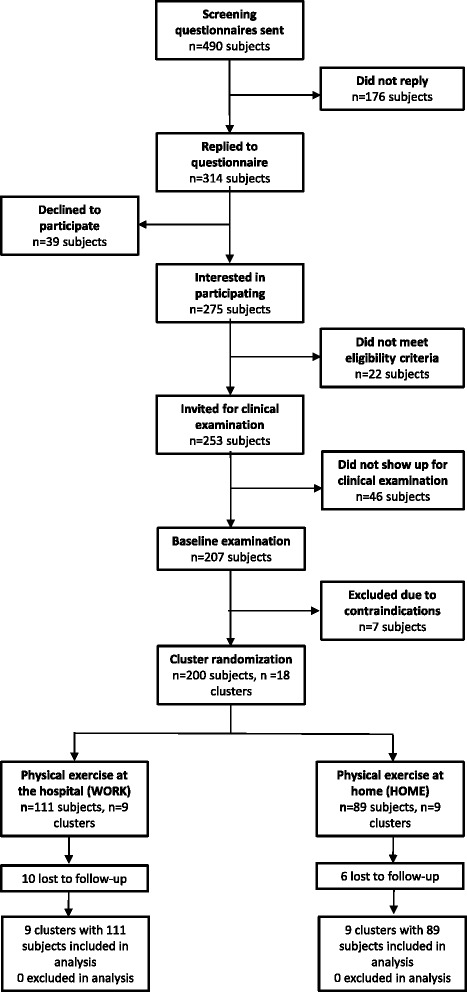
Participant recruitment flow-chart

A screening questionnaire was administered to 490 healthcare workers (aged 18-67 years) from three Danish hospitals in June 2013. Subsequently, in August and September 2013, a total of 207 employees participated in the baseline clinical examination. Exclusion criteria were (1) pregnancy, (2) hypertension (Systolic BP > 160, diastolic BP > 100), (3) a medical history of cardiovascular diseases (e.g. chest pain during physical exercise, heart failure, myocardial infarction and stroke), (4) traumatic or severe injury to the neck, shoulder, arm or hand regions or (5) a medical history of life threatening disease. The overall flow of participant enrolment is depicted in Fig. 1 and has been described in details elsewhere [27].

On the basis of the questionnaire we randomly allocated the 18 departments (200 participants), using a computer-generated random numbers table, to receive either physical exercise at the workplace or at home. The participants at each department and their management were subsequently informed by e-mail about group allocation. At follow-up (i.e. post intervention) testing (Dec 2013-Jan 2014), all examiners were blinded to the group allocation, and participants carefully instructed not to reveal their particular intervention group. Baseline characteristics and work ability scores of the two intervention groups are listed in Table 1. The overall work ability score can be classified into four categories; “poor” (7–27),”moderate” (28–36), “good (37–43) and “excellent” (44–49) work ability [1]. At baseline, 1 %, 9 %, 39 % and 51 % of the entire study population (200 participants) was categorized as having a “poor”, “moderate”, “good” and “excellent” WAI score, respectively.

**Table 1:** Baseline characteristics of the two intervention groups. Values are means (SD)

	Work	Home
N	111	89
Age (years)	40^*^ (12)	44 (10)
Height (cm)	168.4 (6.2)	168.0 (7.2)
Weight (kg)	67.5 (12.1)	68.9 (12.2)
BMI (kg∙m^−2^)	23.8 (3.8)	24.4 (4.0)
Work Ability Index Score (7–49)	42.8 (4,6)	43.3 (4.2)
Item 1: Current work ability compared with the lifetime best (0–10)	8.6 (1.7)	8.7 (1.5)
Item 2: Work ability in relation to the demands of the job (2–10)	8.5 (1.4)	8.8 (1.2)
Item 3: Number of current diseases diagnosed by a physician (1–7)	5.8 (1.3)	6.0 (1.21)
Item 4: Estimated work impairment due to diseases (1–6)	5.7 (0.7)	5.7 (0.8)
Item 5: Sickness absence during the past year (1–5)	3.9 (0.8)	4.1 (0.7)
Item 6: Own prognosis of work ability two years from now (1–7)	6.7 (1.0)	6.7 (1.2)
Item 7: Mental resources (1–4)	3.6 (0.6)	3.5 (0.6)

^*^Denotes difference between groups at baseline, *p* < 0.05. HOME: Home-based physical exercise, WORK: Work-based physical exercise

### Interventions

Participants in each cluster were allocated to a 10-week intervention period receiving either physical exercise at the hospital or physical exercise at home. Both groups were encouraged to perform physical exercises for 5 x 10 min a week. The specific intervention protocols are briefly summarized below, but have been described in detail elsewhere [26].

### Workplace physical exercise (WORK)

Subjects randomized to physical exercise at their workplace (WORK) (*n* = 111 subjects, *n* = 9 clusters) performed group-based and supervised high-intensity strength training using kettlebells, swiss balls and elastic bands (Thera-Band®) during working hours at the hospital. All training sessions took place in designated rooms located at or close to the respective departments and were supervised by an experienced training instructor. In addition, WORK was offered 5 group-based motivational coaching sessions (30–45 min. with 5–12 participants in each session) during working hours.

### Home-based physical exercise (HOME)

Participants randomized to home-based physical exercise (HOME) (*n* = 89 subjects, *n* = 9 clusters) performed physical exercises during leisure time at home. After the participants were informed about group allocation they received a bag with 1) training equipment (easy, medium, and hard elastic tubing) and 2) 3 posters that visually demonstrated the exercises that should be performed for the shoulder-, abdominal- and back muscles, and also contained recommendations for training progression [28–30].

#### Ergonomic training and education

During the period of intervention participants in both groups were offered to participate in brief courses (each of 1½–3 h duration) of ergonomic training and education in patient handling and use of assistive devices. The courses were offered by the hospital’s working environment department who attempted to include all participants in the offered courses during the study period.

### Outcome measures

The outcome measure was the change from baseline to 10-week follow-up in work ability measured by the work ability index score (WAI). WAI provides a composite measure of seven distinct items that aims to capture: 1) current work ability compared with the lifetime best, 2) work ability in relation to the demands of the job (physical and mental demands), 3) number of current diseases diagnosed by a physician, 4) estimated work impairment due to diseases, 5) sickness absence during the last year (12 months), 6) own prognosis of work ability two years from now, and 7) mental resources (worker’s life in general, both at work and during leisure time) [1]. Item 5 (sickness absence during the past year) was categorized into 0 days (5 point), 0 < days < 10 (4 point), 11 ≤ days < 25 (3 point), 26 ≤ days < 100 (2 point), and ≥ 100 days (1 point). Additionally, we explored the contribution of each single item score on the total WAI score by measuring the change in each of the seven items from baseline to follow-up.

### Statistical analysis

All statistical analyses were performed using the SAS statistical software for Windows (SAS Institute, Cary, NC). The change in WAI was evaluated using a repeated-measures two-way analysis of variance (ANOVA) with *group*, *time* and *group by time* as independent variables. Participant was entered as a random effect. Analyses were adjusted for age and WAI at baseline. We performed all statistical analyses in accordance with the intention-to-treat principle using a Mixed model approach which inherently accounts for missing values.

An alpha level of 0.05 is accepted as significant. Outcomes are reported as between-group least mean square differences and 95 % confidence intervals at 10-week follow-up.

Finally, effect sizes were calculated as Cohen’s d [31] (i.e. between-group differences in the WAI scores divided by the pooled standard deviation at baseline). According to Cohen [31], effect sizes of 0.20 are considered small, 0.50 moderate and 0.80 large.

## Results

### Study population

Baseline characteristics of the 200 study participants are shown in Table 1. At baseline, age was slightly higher in HOME compared with WORK (*p* = 0.05). This difference was controlled for by including age as a covariate factor in the statistical analyses. No other between-group differences were observed at baseline.

WORK and HOME, on average, performed 2.2 (SD: 1.1) and 1.0 (SD: 1.2) of the 5 offered training sessions per week, corresponding to a training adherence of 45 % and 21 %, respectively, which differed between groups (*p* < 0.001). In addition, WORK attended on average 2.1 of the 5 offered coaching sessions during the 10-week intervention period. There were no training related injuries reported during the intervention.

### Work ability

**Fig. 2:**
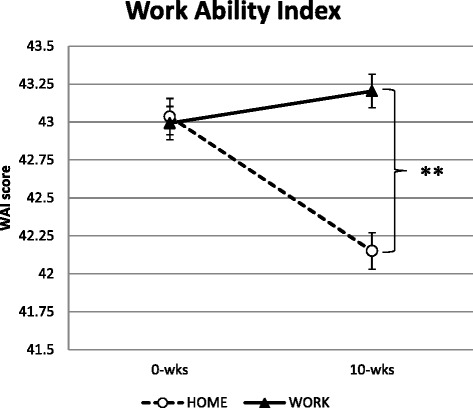
Change in work ability index (WAI) from baseline (0-wks) to follow-up (10-wks) with workplace exercise (WORK; full lines) and home-based exercise (HOME; dashed lines). Values are means (SE) adjusted for baseline values. **Denotes greater reductions in WAI with workplace exercise compared to home-based exercise (Post hoc test: *p* < 0.01)

Figure 2 shows the overall changes in WAI from baseline to 10 week follow-up. A priori hypothesis testing showed a *group by time* interaction for WAI (*p* = 0.03). WAI increased in WORK compared with HOME (Table 2). No within-group change in WAI was observed for participants in WORK (*p* = 0.52) whereas WAI decreased (i.e. worsened) in HOME (*p* = 0.02).

**Table 2:** Changes in work ability index (WAI) and single item scores from baseline to 10-week follow-up

		Work	Home	Work vs Home	
		Difference from 0 to 10 wks	Difference from 0 to 10 wks	Between group difference	*p* Value
WAI score		0.2 (−0.4,0.9)	−0.9 (-1.6,−0.2)	1.1 (0.3,1.8)	0.03^*^
	Item 1: Current work ability compared with the lifetime best (0–10)	0.1 (−0.2,0.4)	−0.1 (−0.4,0.2)	0.2 (−0.1,0.5)	0.31
	Item 2: Work ability in relation to the demands of the job (2–10)	0.3 (0.0,0.6)	−0.1 (−0.4,0.1)	0.3 (0.1,0.6)	0.03^*^
	Item 3: Number of current diseases diagnosed by a physician (1–7)	0.0 (−0.2,0.2)	−0.2 (−0.2,0.0)	0.1 (0.0,0.3)	0.18
	Item 4: Estimated work impairment due to diseases (1–6)	−0.1 (−0.3,0.1)	0.0 (−0.2,0.1)	0.0 (−0.2,0.1)	0.54
	Item 5: Sickness absence during the past year (1–5)	0.1 (0.0,0.3)	−0.1 (−0.2,0.0)	0.2 (0.0,0.3)	0.04^*^
	Item 6: Own prognosis of work ability two years from now (1–7)	0.0 (−0.2,0.2)	−0.2 (−0.4,0.0)	0.3 (0.0,0.5)	0.09
	Item 7: Mental resources (1–4)	−0.2 (−0.3,0.0)	−0.2 (−0.3,0.0)	0.0 (−0.1,0.1)	0.99

^*^Denotes difference between groups at follow-up, *p* < 0.05. HOME: Home-based physical exercise, WORK: Work-based physical exercise. Pre-to-post intervention changes in each group are shown in left columns, and contrasts between the groups are shown in the right hand-side column. Values are means (95 % confidence interval)

Of the seven items of WAI, item 2 (work ability in relation to the demands of the job) and item 5 (sickness absence during the last year (12 months)) increased following WORK compared with HOME (*p* < 0.05; Table 2). No changes in the remainder of the items were observed.

Effect size (Cohen’s d) of the change in WAI score with WORK compared with HOME was 0.24, which was categorized as small-to-moderate (0.20 to 0.50).

## Discussion

The present cluster randomized controlled trial demonstrated that 10 weeks of physical exercise at the workplace was more effective of preventing deteriorations in work ability compared with 10 weeks of home-based exercise in female healthcare workers.

The WAI score can be classified into four categories; “poor” (7–27),”moderate” (28–36), “good (37–43) and “excellent” (44–49) work ability [1]. The baseline average WAI score for our participants was 43.1, which thus could be categorized as “good” or almost “excellent”. This may seem unexpected since almost half of the participants had chronic pain in the lower back, neck and shoulders [27]. In addition, the participants with chronic pain in the neck and shoulders and lower back did demonstrate a slightly lower but still “good” WAI score of 41.9. The relatively high scores of perceived work ability may be explained by the fact that participants were active at the labor market and worked full-time, reflecting a will to sustain work engagement despite the presence of musculoskeletal pain problems.

We rejected the null-hypothesis based on the significant group by time interaction following the 10-week intervention. The group difference was largely driven by a reduction in the WAI score in HOME. The within-group reduction in WAI may not necessarily have been caused by the home-based exercise intervention per se, but may also include the natural seasonal variation in pain symptoms, sickness absence and general health altogether affecting the WAI. Persson and co-workers observed that subjective health complaints peaked from December until February and were lowest during summer among hospital caretakers [32]. Furthermore, Takala and co-workers demonstrated a reduction in neck and shoulder pain symptoms from autumn towards spring among female office workers [33]. In addition, data from the National Health Service (i.e. healthcare personal) in the UK clearly demonstrates seasonal variation in sickness absence during the last 5 years, with peaks in the late autumn/winter months and subsequent reductions towards summer [34]. The above notion may be linked to seasonal affective disorders, which are characterized by low mood, concentration problems, loss of energy and fatigue which are typically increased during autumn and early winter [35]. Thus, the present results in WAI could have been influenced by seasonal variation as baseline testing and questionnaire was collected in August and September and follow-up in December.

We analyzed the seven items of WAI separately and observed a between-group difference in item 2 (work ability in relation to the demands of the job) and item 5 (sickness absence during the last year). The workplace-based exercise resulted in higher gains in back extensors muscle strength,[27] which theoretically may result in lower relative exposure during high-force task as manual patient handling. Lidegaard and co-workers reported lower relative work exposure of the neck muscles in office workers after 10-weeks of strength training [36]. Thus, improved balance between physical work demands and individual physical capacity in WORK compared with HOME may explain the observed improvement of work ability in relation to the demands of the job (item 2).

The effect of physical exercise on sickness absence has been investigated in numerous studies, however, with conflicting results. Svensson and co-workers demonstrated that a multifactorial 14-month prevention program combining physical training, patient transfer technique and stress management reduces self-reported sickness absence compared to a control group among assistant nursing students [37]. However, other multifactorial randomized controlled trials including physical exercise have shown little effect on registered sickness absence among cleaners, construction workers and healthcare workers [19, 38, 39]. Furthermore, a recent Cochrane review concluded that for workers with acute back pain, physical conditioning may not affect sickness absence duration [40]. Moreover, Andersen and co-workers recently demonstrated that a threshold in pain intensity (above 3 on a 0–9 scale) significantly increases the risk for long-term sickness absence among female healthcare workers [41]. Since almost half of our participants reported chronic musculoskeletal pain (≥3 on a 0–10 scale ≥ 3 months) in the neck and shoulder regions and lower back the base for reducing pain and sickness absence were present among this population [27]. Accordingly, along with a reduction in average pain intensity of the neck and shoulder and lower back, workplace-based exercise reduced self-reported sickness absence during the last year (item 5) compared to home-based exercise. Notably, the potential difference in registered and self-reported sickness absence should be taken into account when interpreting the present results.

Numerous studies have investigated the effects of physical exercise on work ability, however, predominantly with little or no effect [18–22]. Low adherence, ranging from 0–2 training sessions per week and high dropout rates may explain the lacking results shown in these studies. Accordingly, Nurminen and co-workers failed to see a change in WAI and sickness absence after performing one year of individually tailored physical exercise (strength training, aerobic training and stretching) once a week among female laundry workers [20]. However, they concluded that perceived work ability and sickness absence cannot be affected very positively using a single-component exercise intervention and argued that work ability promotion may need a more multifactorial approach [20]. The WORK intervention could be seen as a multifactorial approach since including daily supervised training- and motivational coaching sessions. The influence of the specific setting, i.e. exercising together with colleagues at work vs exercising alone at home should be considered in a bio-psychosocial aspect. The bio-psychosocial model focuses on the interaction between biological, psychological, and social factors in the neurological perception of pain [42]. Hence, besides the physiological training effects, the fact that the workplace group performed group-based training- and coaching sessions at the department seemed to have great impact on the social relationship among colleagues and psychological wellbeing of the individual [27]. Accordingly, the positive interplay between the bio-psychosocial factors may have had an additive effect on the perception of WAI in the workplace group.

The present study design aimed at comparing a low cost home-based intervention modality with an intervention protocol that involves investments in training instructors, coaches, working hours and additional exercise equipment. These interventional differences could potentially have influenced training adherence and training intensity. Especially, the higher adherence in WORK compared with HOME may explain our results. By training 2.2 times ten minutes per week the workplace group prevented deterioration of WAI, whereas a decrease in WAI was observed in the home group that trained on average only one time per week. The use of dedicated instructors throughout the intervention may not only ensure proper training intensity and safe exercise execution but also increase participant adherence, since supervised interventions is known to enhance exercise adherence [23]. Furthermore, the provision of 5 coaching sessions for the workplace group may have increased their motivation for attending the daily training and thus increased training adherence.

Implementing longer training sessions may potentially have yielded more positive results. However, the reason for choosing only ten minutes was to offer a low duration, yet effective and realistic training program that could be implemented during the busy working day of a healthcare worker. We were informed by the hospitals health and safety board, prior to the study, that in order to increase training adherence the training sessions should be a short as possible. Thus, although shorter training sessions may compromise the physiological training effect the shorter duration may, however, increase training adherence in this specific hospital setting. Nonetheless, Andersen et al. have previously shown that brief daily sessions of 2 min of shoulder resistance training was equally effective as 12 min sessions in reducing neck and shoulder pain among office workers [43]. However, in order to implement exercises targeting both the neck, shoulder and lower back region we chose to implement 10 min sessions which, when performed twice a week at the workplace, proved not only to be effective in relieving pain intensity but also preventing deterioration of work ability. In line with the adherence and findings observed in WORK, we showed, in a recent study in slaughterhouse workers, that brief sessions (~20 min) of high-intensity strength training performed twice a week at the workplace was effective of preventing a further decline in work ability among workers with chronic musculoskeletal pain [44]. Moreover, Pohjonen & Ranta demonstrated that 9 months of supervised exercise (~1 h. sessions) twice a week improves physical capacity and prevents the decline in work ability after 1 and 5 years in female care aides [21]. Accordingly, performing physical exercise at least twice a week may prevent the age-related deterioration of work ability, health and physical capacity among workers with physically demanding work. However, the small-to-moderate between-group effect size shown in this study indicates that the clinical implications of workplace-based exercise on work ability should be handled with caution.

### Strength and limitations

As perceived work ability may be influenced by outcome expectations a limitation of this study, and behavioral interventions in general, is that blinding of participants and those administrating the intervention was not possible. However, to minimize this type of bias we informed the participants that neither intervention was known to be superior to the other. Moreover, we included two active intervention groups rather than comparing treatment with a waiting list group [45, 46]. A strength of the study is the low loss of participants at follow-up and inclusion of drop-outs in the statistical analysis which allowed us to test the actual effect of the interventions.

## Conclusions

Performing physical exercise at the workplace was more effective of preventing deteriorations in work ability (WAI) compared to home-based exercise in female healthcare workers. This difference in intervention outcome was mainly caused by a reduction in WAI with physical exercise at home. Of the seven items of WAI, item 2 (work ability in relation to the demands of the job) and item 5 (sickness absence during the past year) increased with physical exercise performed at the workplace compared with home-based exercise.
